# Transcriptional profiling of the epigenetic regulator Smchd1

**DOI:** 10.1016/j.gdata.2015.12.027

**Published:** 2015-12-31

**Authors:** Ruijie Liu, Kelan Chen, Natasha Jansz, Marnie E. Blewitt, Matthew E. Ritchie

**Affiliations:** a Molecular Medicine Division, The Walter and Eliza Hall Institute of Medical Research, Parkville, Victoria 3052, Australia; b Department of Medical Biology, The University of Melbourne, Parkville, Victoria 3010, Australia; c School of Mathematics and Statistics, The University of Melbourne, Parkville, Victoria 3010, Australia

**Keywords:** RNA-sequencing, voom, Sample variability, Epigenetics

## Abstract

Smchd1 is an epigenetic repressor with important functions in healthy cellular processes and disease. To elucidate its role in transcriptional regulation, we performed two independent genome-wide RNA-sequencing studies comparing wild-type and *Smchd1* null samples in neural stem cells and lymphoma cell lines. Using an R-based analysis pipeline that accommodates observational and sample-specific weights in the linear modeling, we identify key genes dysregulated by Smchd1 deletion such as clustered protocadherins in the neural stem cells and imprinted genes in both experiments. Here we provide a detailed description of this analysis, from quality control to read mapping and differential expression analysis. These data sets are publicly available from the Gene Expression Omnibus database (accession numbers GSE64099 and GSE65747).

**Table:** 

Specifications	
Organism/cell line/tissue	*Mus musculus*, C57BL/6J strain for lymphoma cell lines, FVB/N/C57BL/6JF1 for neural stem cell lines.
Sex	Male.
Sequencer or array type	NSC data: Libraries prepared with the Illumina TruSeq Total Stranded RNA kit and sequenced on an Illumina HiSeq 2000 with Illumina TruSeq SBS Kit v3-HS reagents as 100 bp paired-end reads. Lymphoma data: Libraries prepared with the Illumina TruSeq RNA Sample Preparation Kit v2 and sequenced on an Illumina HiSeq 2000 with Illumina TruSeq SBS Kit v3-HS reagents as 100 bp reads (paired and single-end).
Data format	Raw (fastq) and summarized counts.
Experimental factors	RNA was obtained from Smchd1 null and wild-type samples.
Experimental features	Neural stem cells were derived from E14.5 male embryos. Lymphoma cells were derived from lethally irradiated mice transplanted with fetal liver cells from E14.5 male embryos at the time when animals for sacrificed due to end-stage lymphoma. The lymphoma cells were plated out for growth in vitro and resulting cell lines analyzed here.
Consent	All animal experiments were carried out in accordance with the Walter and Eliza Hall Institute of Medical Research Animal Ethics Committee guidelines (AEC 2011.027).
Sample source location	Melbourne, Australia.

## 1 Direct link to deposited data

http://www.ncbi.nlm.nih.gov/geo/query/acc.cgi?acc=GSE64099

http://www.ncbi.nlm.nih.gov/geo/query/acc.cgi?acc=GSE65747

## 2 Introduction

Smchd1 (structural maintenance of chromosomes hinge domain containing 1) is an important epigenetic modifier that has a critical role in X inactivation [1], [2] and genomic imprinting [3], [4]. Although initial studies of Smchd1 used these two classic models of epigenetic control, it has become clear that Smchd1 has a broader role in regulating gene expression during normal development [5], in cancer [6] and in the development of facioscapulohumeral muscular dystrophy (FSHD) [7], [8], [9].

We were particularly interested to look at the role of Smchd1 in regulating gene expression via RNA sequencing (RNA-seq), as Smchd1 is a repressor protein, and so the very low level of expression of Smchd1 repressed genes best lends itself to RNA-seq over array-based platforms. To this end, we conducted RNA-seq experiments in two model systems, the first was in neural development using neural stem cells and the second was in a cancer model using lymphoma cell lines. In both experiments, samples with wild-type levels of Smchd1 are compared to samples with a null allele of this gene. This article describes our analyses of these two data sets, using a consistent, R-based pipeline that can deal with both observational and sample-level heterogeneity.

## 3 Experimental design, materials and methods

### 3.1 Mouse strains and sample information

MommeD1 mutant mice were maintained on the FVB/N inbred background, and backcrossed with C57BL/6 mice for more than 15 generations to produce C57BL/6 MommeD1 congenic mice (as previously described in [1]). Neural stem cells were isolated and cultured from the brains of FVB/C57BL/6J F1 E14.5 male embryos, homozygous or wild-type for the *Smchd1*^MommeD1^ mutation as described in [5]. Lymphoma cell lines were derived from a gene trap allele of Smchd1, described in [6]. This allele was backcrossed onto C57BL/6J, then crossed onto the Eμ-Myc transgenic background to generate *Smchd1*^*gt*/*gt*^ *Eμ-MycTg*/*+* embryos and their wild-type controls, for transplant and generation of lymphomas. Genotyping was carried out as described in [1], [2] and [6]. Experimental animals were treated in accordance with the Australian Government National Health and Medical Research Council guidelines under the approval from the animal ethics committees of the Walter and Eliza Hall Institute (WEHI AEC 2011.027).

### 3.2 RNA-seq sample preparation and sequencing

Qiagen RNeasy Mini kits were used to extract RNA from *Smchd1*^MommeD1/MommeD1^ and *Smchd1*^*+*/*+*^ wild-type NSCs according to the manufacturer's instructions. RNA was quantified using the NanoDrop 1000 Spectrophotometer (Thermo Scientific) and RNA integrity assessed with the Agilent Bioanalyzer 2100 (Agilent Technologies). Illumina's TruSeq total RNA sample preparation kit was used to prepare libraries for sequencing, which was performed by the Australian Genome Research Facility (Melbourne, Australia) on the Illumina HiSeq 2000 platform to obtain 100 bp paired-end reads.

For the Lymphoma data set, Qiagen RNeasy Mini kits were used to extract RNA from *Smchd1*^*MommeD1*/*MommeD1*^;*E*μMycTg/*+* and *Smchd1*^*+*/*+*^;*E*μMycTg/*+* lymphoma cells. Samples were prepared for sequencing at the Australian Genome Research Facility where quality control, library preparation (using Illumina's TruSeq RNA sample preparation kit) and sequencing on the Illumina HiSeq 2000 platform was performed to obtain 100 bp paired-end (for 6 out of 7 samples) or single-end (for 1 sample) reads.

### 3.3 Quality control and data pre-processing

**Fig. 1:**
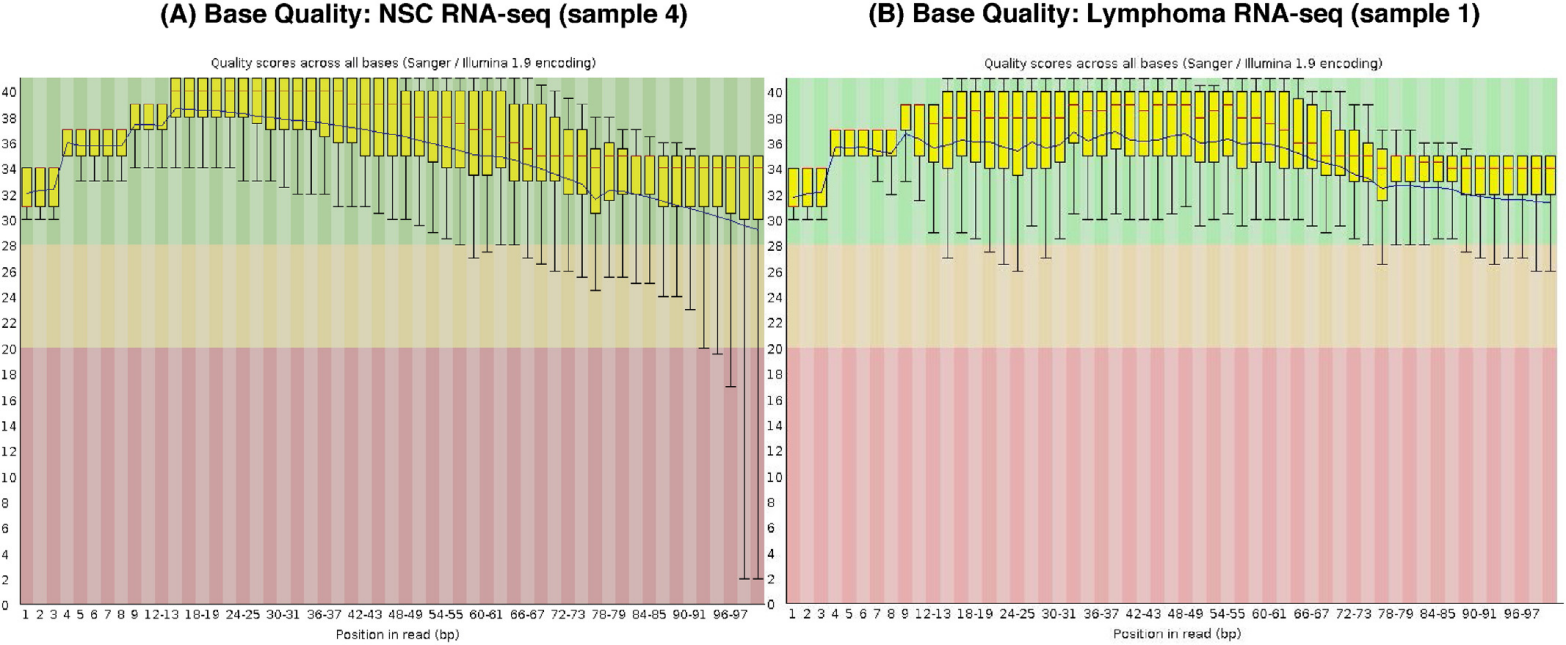
Quality assessment at the read level. Boxplots of base-calling Phred scores at different base positions across all the reads in representative libraries from NSC RNA-seq (A) and Lymphoma cell line RNA-seq (B) experiments generated by FastQC. The box represents 25% and 75% quantiles of the scores with median score marked by the red line. Whiskers mark the 10% and 90% quantiles and blue lines show the mean quality score.

The FastQC software [10] was used to assess the quality of the raw sequence data. Fig. 1 displays the distribution of sequencing quality (Phred) scores at each base position across reads from a representative RNA-seq sample from each data set. Although variation in base quality is observed across the read, with slightly lower quality at the beginning and end, median quality is above 34 (corresponding to a probability of an incorrect base call below 0.0004) for the entire read. Similar boxplots of base quality scores were observed for other samples (data not shown).

Sequences were then mapped to the mouse reference genome (mm10) using the *Rsubread* program [11] and gene-level counts were obtained by the *featureCounts* procedure [12].

**Fig. 2:**
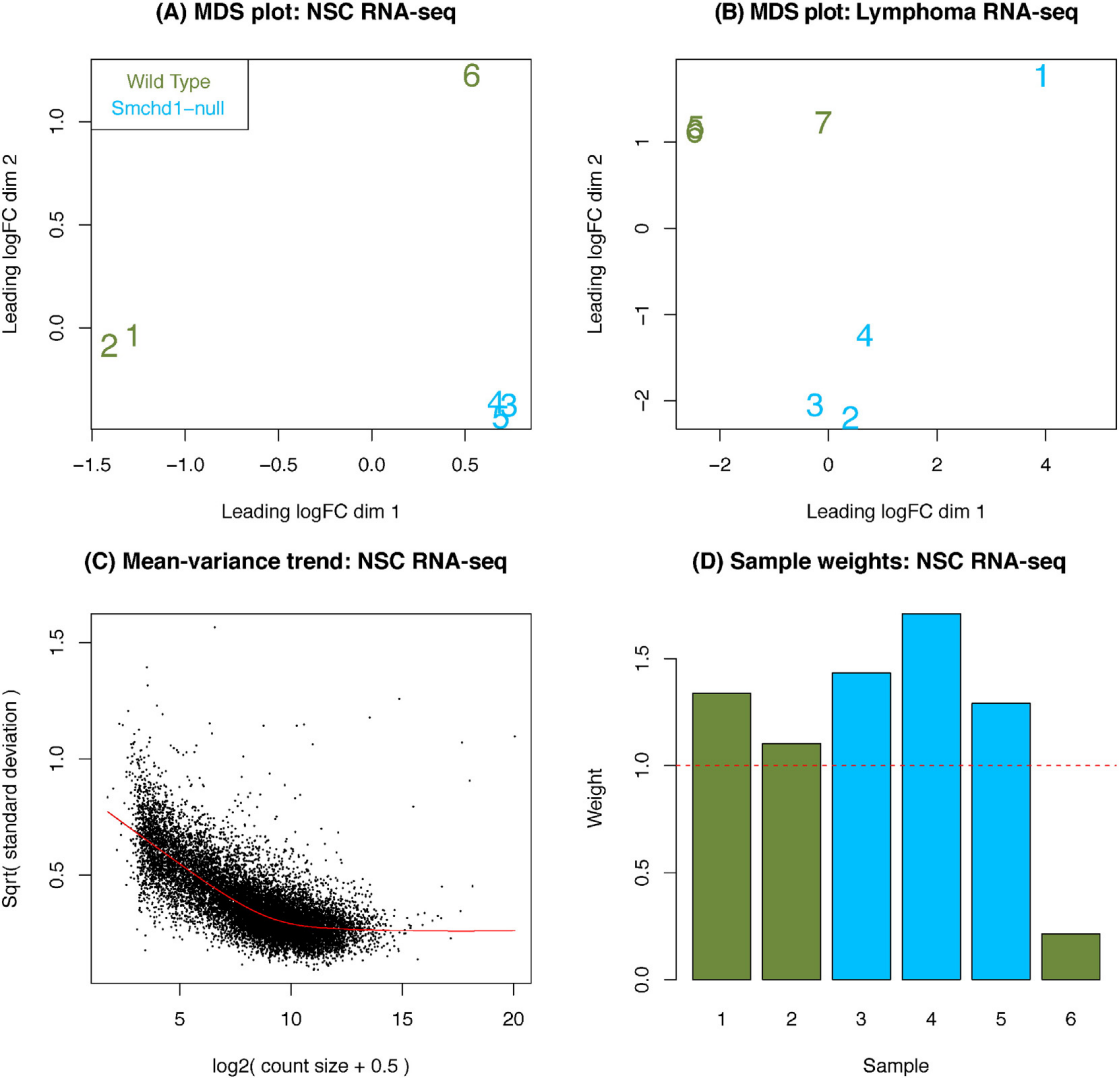
Quality assessment at the sample level. Multi-dimensional scaling (MDS) plots of the NSC (A) and Lymphoma (B) data sets, with samples numbered and color coded by genotype. Distances correspond to the mean log_2_fold-change for the top 500 genes that best discriminate each pair of samples. In both experiments, one or more samples cluster poorly with replicates of the same genotype, motivating the use sample weights (D) in the regression modeling to detect differential expression. Panel C shows a scatterplot of the mean–variance relationship in abundance estimated from biological replicates from the NSC data set using the *voom* method. Panel D shows the sample weights estimated for the NSC data set that are combined with *voom*'s abundance-related weights in the *voomWithQualityWeights* function and used in the linear model analysis to detect differentially expressed genes.

Further analysis was carried out using the *edgeR* [13] and *limma* [14] R/Bioconductor packages. Counts-per-million (CPM) were calculated for each gene to standardize for differences in library-size and filtering was carried out to retain genes with a baseline expression level of at least 0.5 CPM in 3 or more samples. For each data set, TMM normalization [15] was applied and a multidimensional scaling (MDS) plot based on the log_2_(CPM) was generated to show relationships between samples (Fig. 2). In both data sets, we observe samples that do not cluster well with their respective replicates of the same genotype. Sample 6 in the NSC data (Fig. 2A) and samples 1 and 7 in the Lymphoma data (Fig. 2B) are more variable than the other replicates of the same type. For NSC sample 6 and Lymphoma sample 7, there was no experimental factor that could be identified to explain this phenomenon. Lymphoma sample 1 on the other hand was the only single-end sample in this experiment that was processed on a different day to the other samples, leading us to conclude that batch processing differences was the likely cause of the additional variation.

### 3.4 Differential expression analysis

Based on inspection of the MDS plots, which showed variability between replicate samples, linear models [16] with combined observational and sample weights [17], [18] were fitted to the log_2_(CPM) to summarize over replicate samples. This strategy, implemented in the *voomWithQualityWeights* function, down-weights low abundance observations, which are systematically more variable (Fig. 2C) and observations from entire samples that show higher variation (Fig. 2D) to get more precise estimates of gene expression and increase power to detect changes. Moderated *t*-statistics were used to assess differential expression between *Smchd1*^*+*/*+*^ wild-type and *Smchd1*^MommeD1/MommeD1^ samples, with genes ranked according to their false discovery rate [19]. Log-odds of differential expression [20] were also calculated. Both raw and summary-level count data for these experiments are available under GEO series accession numbers GSE64099 and GSE65747.

## 4 Results

**Fig. 3:**
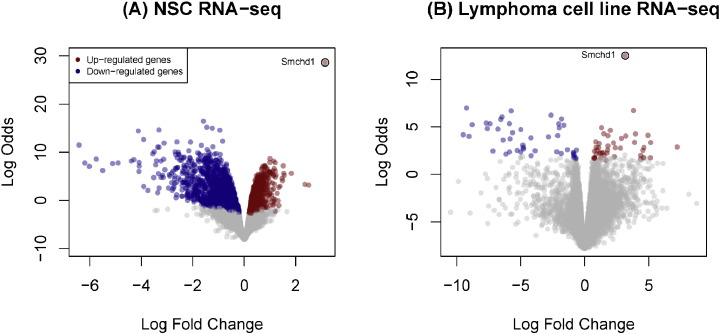
Summary of the RNA-seq results. Volcano plot representation of differential expression analysis of genes in the *Smchd1* wild-type versus *Smchd1* null comparison for the NSC (A) and Lymphoma RNA-seq (B) data sets. Red and blue points mark the genes with significantly increased or decreased expression respectively in *Smchd1* wild-type compared to *Smchd1* null samples (FDR < 0.01). The x-axis shows log_2_fold-changes in expression and the y-axis the log odds of a gene being differentially expressed. In both data sets, *Smchd1* is the top ranked gene.

At a false discovery rate (FDR) cut-off of 1%, there are 2838 differentially expressed genes (1282 up-regulated and 1556 down-regulated) in the comparison of *Smchd1* wild-type and *Smchd1* null NSC samples. The same comparison in the Lymphoma data set detected 90 genes (45 up-regulated and 45 down-regulated). These genes are highlighted in Fig. 3A and B respectively. In both analyses, *Smchd1* is the top ranked gene with log_2_fold-change greater than 3.1.

The NSC analysis revealed that a number of protocadherin genes, especially those from the alpha and beta clusters, were significantly differentially expressed, with down-regulation of 11 alpha cluster genes and 20 beta cluster genes. This finding is in line with studies performed in other tissues and cell lines where Smchd1-deficiency is concomitant with increased expression of protocadherin genes [3], [4], [6]. However, the widespread impacts observed in this analysis suggest that Smchd1 plays a critical role in regulating the protocadherin clusters in NSCs. Imprinted genes, such as *Ndn*, *Mkrn3* and *Peg12* were down-regulated by almost 2-fold, indicative of loss of imprinting in the absence of Smchd1, also in agreement with results of previous studies [3], [4], [6].

Genes uncovered in the Lymphoma analysis are consistent with previous reports in a different system that profiled male embryos [2], where the expression of imprinted genes such as *Peg12* and *Mkrn3* was shown to be disturbed in the absence of Smchd1. However it is interesting to note that *Peg12* and *Mkrn3* are much more strikingly down-regulated in the Lymphoma data set than in the NSCs as they are normally only very lowly expressed in the lymphoma cell lines. This may represent not just loss of imprinting, as has been shown previously [2], [3], but also potential activation independent of imprinting status.

The modest number of differentially expressed genes identified in the Lymphoma data set is influenced in part by a suspected batch processing difference mentioned earlier, but also by the increased genetic heterogeneity present in profiles obtained from tumor samples. In contrast, many more genes are detected in the NSC experiment, where the samples are genetically equivalent and much less heterogeneous and genetically unstable than the lymphoma cell lines.

## 5 Discussion

In this report we provide a detailed description of the analysis of the RNA-seq data from [5], [18] made possible using an R-based processing pipeline in the *Rsubread* and *limma* packages. In particular, the *voomWithQualityWeights* function in *limma* allows more variable samples to be down-weighted in the analysis [18]. In each case, the decision to use this approach was guided by inspection of the MDS plot to assess how well replicate samples clustered. This methodology is generally applicable to analyses of designed RNA-seq experiments, where variations in sample quality are frequently observed and the source of such variation is generally unknown. Scripts and data to reproduce this analysis are available from http://bioinf.wehi.edu.au/folders/smchd1/.
